# Ribosomal intergenic spacer (IGS) length variation across the Drosophilinae (Diptera: Drosophilidae)

**DOI:** 10.1186/1471-2148-5-46

**Published:** 2005-08-19

**Authors:** Mariana Mateos, Therese A Markow

**Affiliations:** 1 Center for Insect Science and Department of Ecology and Evolutionary Biology, University of Arizona, BioSciences West 310, Tucson, AZ 85721, USA

## Abstract

**Background:**

The intergenic spacer of the ribosomal genes in eukaryotes (IGS) contains duplications of the core transcription promoter. The number of these duplicated promoters, as measured by the IGS length, appears to be correlated with growth rate and development time in several distantly related taxa. In the present study, we examined IGS length variation across a number of species of *Drosophila *to determine the amount of variation in this trait across different evolutionary time scales. Furthermore, we compared the usefulness of two methods commonly used to determine IGS length: Southern Blot Hybridization (SB) and Polymerase Chain Reaction (PCR).

**Results:**

Our results show broad variation in IGS length across the genus *Drosophila*, but closely related species had similar IGS lengths. Our results also suggest that PCR tends to underestimate the true IGS size when the size is greater than 5 kb, and that this degree of underestimation is greater as the IGS size increases.

**Conclusion:**

Broad variation in IGS length occurs across large evolutionary divergences in the subfamily Drosophilinae. Although average IGS length has been shown to evolve rapidly under artificial selection, closely related taxa generally have similar average IGS lengths. Our comparison of methods suggests that without previous knowledge of the DNA sequence of the IGS and flanking regions, both methods be used to accurately measure IGS length.

## Background

Due to the importance of ribosomes in protein synthesis, cellular growth, and organismal development, ribosomal genes are highly transcribed; with ribosomal RNA accounting for nearly half of all cellular transcription and 80% of the RNA content of growing cells [reviewed by [1,2]]. To achieve these high levels of ribosome production, eukaryotes have multiple copies of ribosomal (r)DNA, arranged in tandem in the Nucleolus Organizer Regions (NORs) of one or more chromosomes. In addition, eukaryotes sustain high levels of transcription per rDNA copy [1].

**Figure 1:**
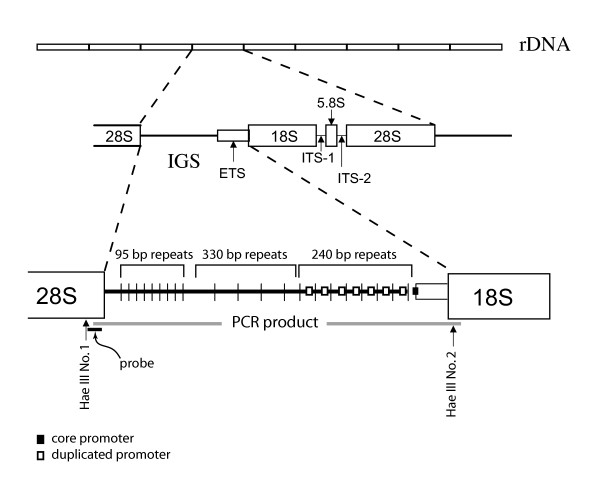
**Ribosomal DNA array in *Drosophila melanogaster***. Diagram shows position of PCR product and primers, *Hae *III restriction sites at the ends of the intergenic spacer (IGS) region, and hybridization probe used in this study [modified from 9, 14].

The structure of the ribosomal intergenic spacer (IGS; Figure 1) appears to be important for achieving these high transcription levels. IGS varies in length from about 2 kb in yeast to 21 kb in mammals, and is also highly variable among and even within individuals of the same species [reviewed by [1]]. These length polymorphisms are mostly due to variation in the numbers of different internal subrepeats present in the IGS (Figure 1). In eukaryotes, some of these repetitive regions contain duplications of the core promoter [reviewed by [1]]. These promoter duplications have been shown to enhance rDNA transcription. For example, in *Drosophila melanogaster*, activity of the rDNA promoter is directly correlated with the number of IGS subrepeats that contain a promoter duplication [3,4]. Selection studies also support the idea that IGS structure is important for rDNA transcription and consequently for growth rate. Cluster et al. [5] found a relationship between IGS length and development time in *D. melanogaster*, where lines selected for fast development had on average, longer IGS variants (attributed to more copies of the promoter duplication) than lines selected for slow development. Similarly, under selection for rapid growth rate, average IGS length increased in *Daphnia pulex *[6], and after selection for high yield, the frequency of long spacers increased in maize [7]. Furthermore, longer spacers are associated with higher growth rates in several species of *Daphnia *[8]. Although these studies suggest that IGS length may have significant effects on life history traits, the evolutionary significance of IGS length remains a mystery. An initial step to understanding the evolutionary role of IGS length is to characterize its variation across a group of related taxa that could then be used to test hypotheses about the adaptive significance of IGS length using the comparative method. The main goal of the present paper is to characterize IGS length variation across a wide range of *Drosophila *species (subfamily Drosophilinae) and determine the amount of variation observed across different evolutionary time scales.

Studies of IGS length variation have commonly relied on Southern Blot hybridization (SB) for inference of IGS length. However, more recently, several studies have used PCR to determine IGS length [6,8-10]. Each of these methods has advantages and disadvantages. The main disadvantage of PCR is that certain fragments (particularly the smaller ones) may be selectively amplified, and that it may not amplify large (> 4 kb) fragments. Thus, the amplified products may not represent the actual size frequency distribution of the IGS. The main disadvantage of SB is that it requires more DNA to begin with. Both methods require previous knowledge about the DNA sequence; either for the design of primers for PCR or for the selection of restriction digestion enzymes for Southern blot hybridization. The secondary goal of this study is to compare the usefulness of each method in estimating IGS length across a group of related taxa when knowledge about sequence of IGS and flanking regions is restricted to a small subset of the taxa under examination. Therefore, design of PCR primers and selection of restriction enzymes is based on this limited number of sequences.

## Results

We examined IGS length variation based on Southern Blot hybridization SB and/or PCR in 71 species of the Drosophilinae representing 20 species groups in the genus *Drosophila *and four other genera (Table 1). Of these, only 52 yielded PCR product. Therefore, for the remaining 19 species we were able to infer IGS length based only on SB. Of the 52 species for which we obtained PCR product, 29 (representing 11 species groups in two subgenera of the genus *Drosophila*) had a single restriction site near one of the ends. This was the restriction digestion pattern we expected based on the *D. melanogaster *map (i.e., *Hae *III site No. 2; Figure 1). From the remaining 23 species, nine did not have a restriction site within the PCR fragment; thus SB-based inferences of IGS length in these nine species would have overestimated the true IGS length. In contrast, the remaining 14 species had more than one restriction site within the PCR fragment. Therefore, SB-based inferences of IGS length in these 14 species would have underestimated the true IGS length.

**Table 1:** IGS length results for each of the species examined in this study. IGS size index, IGS size range, three most dominant IGS sizes (% relative proportion), and number of bands. Presence and distance of the *Hae *III restriction site(s) from the end of PCR-amplified IGS products are indicated for each species.

**Species**	**Size Index**	**Size Range**	**Size of lost fragments (bp) after digestion of PCR products with *Hae*III**	**Dominant 1**	**Dominant 2**	**Dominant 3**	**No. of bands**	**No. of females**	**Strain ID or source locality**
**Based on Southern Blot Hybridization (SB) and PCR**									
Subgenus *Sophophora*									
Species group: *melanogaster*									
*D. yakuba *(SB)	5.30	2.63–7.76		5.22 (33.2)	6.98 (15.9)	4.6 (13.0)	12	9	14021-0261.0
*D. yakuba*(PCR)	3.88	2.68–4.64	150	4.64 (30.5)	4.01 (23.4)	3.62 (21.7)	6	1	14021-0261.0
*D. melanogaster *(SB)	6.37	3.87–12.89		5.5 (17.6)	5.2 (13.8)	6.9 (11.4)	15	1	Tucson, AZ
*D. melanogaster *(SB)	5.94	2.64–14.79		5.22 (11.8)	4.89 (10.2)	5.66 (9.0)	19	6	Tucson, AZ
*D. melanogaster *(SB)	5.73	5.08–7.84		5.62 (54.7)	5.36 (21.2)	6.29 (10.2)	9	1	Tucson, AZ
*D. melanogaster*(PCR)	2.72	1.98–3.11	240	3.11 (63.2)	2.12 (23.7)	1.98 (13.2)	3	1	Tucson, AZ
*D. eugracilis *(SB)	3.64	2.37–7.32		4.28 (22.8)	3.94 (15.0)	4.61 (10.4)	12	5	14028-0451.3
*D. eugracilis*(PCR)	4.83	4.83	470, 170	4.83 (100)			1		14028-0451.3
*D. varians *(SB)	7.49	7.44–8.24		7.44 (93.2)	8.24 (6.8)		2	8	14024-0431-0
*D. varians*(PCR)	3.11	2.64–3.96	210	2.64 (64.2)	3.96 (35.8)		2	1	14024-0431-0
*D. kikkawai *(SB)	5.19	3.18–11.38		4.8 (17.6)	4.36 (13.1)	4.61 (9.6)	17	7	14028-0561.0
*D. kikkawai*(PCR)	4.21	3.55–4.89	435	4.48 (29.5)	3.55 (26.4)	4.89 (23.5)	4		14028-0561.0
*D. auraria *(SB)	4.81	3.5–4.91		4.91 (90.6)			4	7	14028-0471.0
*D. auraria*(PCR)	2.86	2.86	520	2.86 (100)			1	1	14028-0471.0
*D. kanapiae *(SB)	5.69	5.58–5.78		5.78 (54.9)	5.58 (45.1)		2	6	14028-0541.0
*D. kanapiae*(PCR)	5.44	4.73–5.95	540	5.95 (58.1)	4.73 (41.9)		2	1	14028-0541.0
*D. parvula *(SB)	4.48	3.44–6.35		5.14 (18.9)	4.88 (17.9)	4.4 (14.2)	11	6	14028-0621.0
*D. parvula*(PCR)	5.46	5.46	620	5.46 (100)			1	1	14028-0621.0
									
Species group: *obscura*									
*D. pseudoobscura *(SB)	3.30	1.68–4.27		3.65 (30.2)	3.36 (25.4)	3.93 (11.5)	10	10	TE 1198-2
*D. pseudoobscura*(PCR)	3.30	3.30	150	3.30 (100)			1	5	TE 1198-2
*D. persimilis *(SB)	3.35	1.94–4.5		3.67 (17.0)	3.34 (16.5)	3.02 (15.1)	10	3	14011-0111.24
*D. persimilis*(PCR)	3.23	2.71–3.99	150	3.40 (36.1)	3.11 (23.9)	2.90 (18.6)	6	5	14011-0111.24
*D. miranda *(SB)	4.54	3.78–5.29		4.86 (36.4)	4.56 (28.6)	4.28 (18.3)	6	5	14011-0101.11
*D. miranda*(PCR)	3.78	2.93–4.45	180	4.14 (17.5)	3.85 (17.4)	3.54 (16.0)	7	1	14011-0101.11
*D. affinis *(SB)	4.73	2.82–7.46		4.32 (10.4)	4.75 (10.2)	5.08 (10.02)	16	6	14012-0141.0
*D. affinis*(PCR)	3.43	2.93–4.11	180	3.14 (29.6)	3.45 (18.8)	2.93 (18.1)	6		14012-0141.0
*D. bifasciata *(SB)	5.54	3.19–8.68		5.58 (9.7)	5.24 (9.2)	4.91 (8.9)	15	3	14012-0181.1
*D. bifasciata*(PCR)	3.62	2.84–4.77	200	3.76 (18.2)	3.29 (16.5)	3.54 (16.2)	8	1	14012-0181.1
									
Species group: *willistoni*									
*D. paulistorum *(SB)	11.60	7.08–16.56		10.53 (34.1)	11.67 (33.4)	15.67 (13.6)	6	8	14030-0771.11
*D. paulistorum*(PCR)	3.55	3.38–3.82	340	3.38 (62.0)	3.82 (38.0)	2.87 (7.2)	2	1	14030-0771.11
*D. equinoxialis *(SB)	6.77	5.4–10.04		6.22 (34.2)	7.93 (18.3)	6.9 (17.7)	6	7	14030-0741.0
*D. equinoxialis*(PCR)	2.74	2.52–3.07	170	2.52 (57.9)	3.07 (34.9)		3	1	14030-0741.0
*D. willistoni *(SB)	5.90	5.65–6.5		5.65 (70.4)	6.5 (29.6)		2	7	14030-0811.3
*D. willistoni*(PCR)	1.32	1.31–3.07	200	1.31 (99.0)	2.59 (0.3)	1.52 (0.3)	4	1	14030-0811.3
									
Subgenus *Drosophila*									
Species group: *virilis*									
*D. novamexicana *(SB)	5.11	3–8.18		5.33 (16.9)	6 (16.1)	4.96 (15.9)	11	5	15010-1031.4
*D. novamexicana*(PCR)	4.33	1.51–5.11	200	5.11 (42.0)	4.70 (41.0)	1.51(17.0)	4	1	15010-1031.4
*D. lummei *(SB)	5.56	4.88–7.64		5.53 (56.7)	5.17 (19.5)	4.88 (7.5)	6	4	15010-1011.2
*D. lummei*(PCR)	4.74	3.73–4.97	190	4.97 (70.2)	4.47 (15.4)	3.73 (5.0)	5	1	15010-1011.2
*D. virilis *(SB)	5.53	2.53–7.39		5.5 (27.7)	6.08 (21.6)	4.86 (16.2)	15	3	15010-1051.12
*D. virilis*(PCR)	1.57	1.42–3.56	110	1.56 (60.5)	1.45 (39.2)		3	1	15010-1051.12
*D. kanekoi *(SB)	8.01	6.37–8.9		8.15 (47.1)	7.33 (30.1)	8.9 (20.2)	4	1	15010-1061.0
*D. kanekoi*(PCR)	6.75	6.04–7.60	185	6.04 (45.4)	7.60 (54.6)		2	1	15010-1061.0
									
Species group: *repleta*									
*D. nigrospiracula *(SB)	5.25	4.75–6.17		4.75 (34.9)	5.65 (33.8)	5.02 (14.2)	5	1	15081-1503.1
*D. nigrospiracula*(PCR)	4.32	3.35–5.28	290, 210	5.28 (25.4)	4.04 (25.2)	3.78 (25.0)	6	1	15081-1503.1
									
Species group: *dreyfusi*									
*D. camargoi *(SB)	5.01	3.51–8.21		4.57 (11.01)	4.33 (10.9)	3.94 (10.1)	12	3	15060-1221.2
*D. camargoi *(PCR)	3.74	2.90–4.32	150	4.32 (21.64)	3.76 (16.98)	4.00 (16.3)	7	1	15060-1221.2
									
Species group: *mesophragmatica*									
*D. gaucha *(SB)	6.67	5.06–8.18		6.05 (21.6)	6.64 (21.5)	7.25 (21.2)	5	4	15070-1231.0
*D. gaucha*(PCR)	4.41	2.49–5.23	160	2.49 (41.9)	5.23 (69.1)		2	1	15070-1231.0
									
Species group: *nannoptera*									
*D. pachea *(SB)	9.00	6.98–14.35		8.04 (32.4)	6.98 (25.2)	8.76 (16.6)	6	7	Ejido
*D. pachea*(PCR)	7.89	7.89	620	7.89 (100)			1	1	Ejido
*D. nannoptera *(SB)	4.90	3.35–11.5		3.35 (23)	7.22 (12.7)	3.94 (11.8)	13	6	15090-1692.2
*D. nannoptera*(PCR)	4.12	3.39–4.70	670	3.39 (26.3)	4.70 (24.5)	4.27 (22.1)	5	1	15090-1692.2
									
Species group: *immigrans*									
*D. nasuta *(SB)	6.30	5.16–7.09		6.49 (40.0)	6.13 (24.8)	7.09 (13.5)	6	1	15112-1781.9
*D. nasuta*(PCR)	2.98	2.79–3.67	250	3.67 (74.9)	3.49 (19.1)	3.67 (6.0)	3	1	15112-1781.9
*D. albomicans *(SB)	6.54	2.36–10.48		7.22 (14.5)	8.89 (13.28)	6.73 (13.18)	12	10	15112-1751.2
*D. albomicans*(PCR)	1.34	1.20–1.61	240	1.61 (67.3)	1.20 (13.0)	1.29 (7.6)	3	1	15112-1751.2
									
Species group: *tripunctata*									
*D. tripunctata *(SB)	4.62	1.4–10		6.47 (14.3)	5.61 (13.2)	7.51 (12.3)	8	4	15220-2401.2
*D. tripunctata*(PCR)	4.12	2.98–5.32	180	3.83 (36.4)	5.32 (24.8)	3.71 (19.1)	6	1	15220-2401.2
									
Species group: *testacea*									
*D. putrida *(SB)	3.73	3.73		3.73 (100)			1	7	15150-2101.1
*D. putrida*(PCR)	3.84	3.84	590	3.84 (100)			1	1	15150-2101.1
									
**Based on PCR only**									
Subgenus *Sophophora*									
Species group: *melanogaster*									
*D. ananassae*	4.15	4.15	220, 830	4.15 (100)			1	1	14024-0371.3
*D. pallidosa*	4.33	3.16–4.82	400, 520, 860	4.17 (78.5)	4.82 (24.9)		3	1	14024-0433.1
*D. greeni*	2.72	2.27–4.18	160, 1066	2.27 (65.5)	4.18 (20.9)	2.51 (7.1)	4	1	14028-0712.0
*D. seguyi*	2.40	2.40	842, 490, 450	2.40 (100)			1	1	14028-0671.0
*D. lini*	3.38	3.33–3.64	120, 710, 1700, 1880, 2080	3.33 (81.2)	3.64 (18.8)		2	1	14028-0581.0
*D. mayri*	3.65	3.54–3.72	130, 210, 440	3.72 (60.1)	3.54 (39.8)		2	1	14028-0591.0
*D. birchii*	2.42	1.88–2.93	no *Hae *III site	2.93 (49.6)	1.98 (25.1)	1.88 (25.3)	2	1	14028-0521.0
*D. baimaii*	4.65	3.38–5.49	500, 640	4.92 (51.9)	3.38 (25.7)	5.49 (22.4)	3	1	14028-0481.1
									
Subgenus *Dorsilopha*									
*D. busckii*	4.04	3.6–4.87	1547, 1016	3.60 (65.5)	4.87 (34.5)		2	1	Anza Borrego, CA
									
Subgenus *Drosophila*									
Species group: *repleta*									
*D. bifurca*	4.77	2.90–5.91	140, 190, 900	5.91 (44.7)	4.64 (29.2)	2.90 (26.1)	3	1	15085-1621.0
*D. mojavensis*	4.21	3.25–7.09	no *Hae *III site	3.72 (37.0)	4.00 (19.9)	4.99 (9.1)	6	1	San Carlos, Son., Mexico
*D. mojavensis*	4.81	4.81	no *Hae *III site	4.81 (100)			1	1	Ensenada de los Muertos, B.C.S., Mexico
*D. arizonae*	4.31	4.05–5.39	no *Hae *III site	4.05 (80.7)	5.39 (19.3)		2	1	Tucson, AZ
*D. mettleri*	7.09	7.09	220, 370, 750, 2950, 3250	7.09 (100)			1	1	Organ Pipe National Monument, AZ
*D. aldrichi*	5.47	5.47	no *Hae *III site	5.47 (100)			1	1	Tucson, AZ
									
Species group: *bromeliae*									
*D. bromeliae*	4.39	1.63–4.50	200, 250, 440, 1640	4.50 (75.4)	4.05 (24.6)		4	1	10585-1682.0
									
Species group: *funebris*									
*D. funebris*	4.20	3.93–4.36	no *Hae *III site	4.36 (76.1)	3.93 (23.9)		2	1	15120-1911.3
									
Species group: *calloptera*									
*D. ornatipennis*	4.33	3.29–5.00	210, 320, 630	5.00 (45.3)	3.29 (31.3)	4.72 (23.4)	3	1	15160-2121.0
									
Species group: *cardini*									
*D. dunni thomasiensis*	4.04	2.27–4.31	150, 560, 1000, 1060	4.03 (49.1)	3.85 (34.2)	4.31 (24.0)	4	1	15182-2301.0
*D. parthenogenetica*	3.54	3.33–4.16	no *Hae *III site	3.33 (53.3)	3.53 (27.4)	4.16 (19.3)	3	1	15181.2221.0
									
Other genera									
*Scaptodrosophila lebanensis*	6.13	6.13	no *Hae *III site	6.13 (100)			1	1	11020-0021.0
*Hirtodrosophila duncani*	5.03	4.23–6.07	no *Hae *III site	4.23 (35.9)	6.07 (34.0)	4.75 (30.0)	3	1	92000-0075.0
*Zaprionus ghesquerei*	4.98	3.52–8.45	880, 600, 290	5.31 (35.7)	6.35 (16.6)	3.51 (15.2)	6	1	50000-2743.0
									
**Based on Southern Blot Hybridization (SB) only**									
Subgenus *Sophophora*									
Species group: *melanogaster*									
*D. teissieri*	4.86	4.6–6.08		4.75 (58.3)	5.04 (23.7)	4.6 (13.5)	4	5	14021-0257.0
*D. teissieri*	5.20	4.95–6.27		4.95 (58.4)	5.22 (28.6)	6.27 (12.9)	3	12	14021-0257.0
*D. barbarae*	5.61	3.31–10.68		4.78 (30.3)	6.2 (18.5)	5.26 (14.1)	10	10	14028-0491.1
*D. punjabiensis*	7.16	4.14–8.64		7.24 (15.3)	7.68 (14.2)	8.05 (14.0)	11	7	14028-0641.0
*D. bicornuta*	10.77	4.88–16.67		11.91 (56.8)	9.67 (15.7)	8.57 (11.6)	6	6	14028-0511.0
									
Subgenus *Drosophila*									
Species group: *virilis*									
*D. montana*	6.42	1.39–18.83		10.55 (9.8)	8.38 (9.8)	6.69 (9.4)	16	4	15010-1021.24
*D. borealis*	7.84	5.38–8.8		7.82 (46.5)	7.23 (26.6)	8.8 (23.7)	5	4	15010-0961.0
									
Species group: *repleta*									
*D. hydei*	5.16	3.44–8.66		4.5 (18.3)	5.88 (17.3)	4.81 (17.0)	10	3	15085-1641.28
*D. navojoa*	7.18	3.1–11.73		6.7 (26.63)	7.18 (20.2)	6.08 (14.6)	13	5	15081-1374.0
*D. micromettleri*	5.67	4.1–11.55		5.15 (38.0)	6.2 (35.7)	5.52 (11.0)	8	7	15081-1346.0
*D. eremophila*	5.02	2.45–13.47		4.9 (23.1)	5.91 (13.5)	7.06 (11.4)	13	6	15081-1292.0
*D. wheeleri*	7.18	6.47–9.76		7.03 (21.9)	6.69 (21.4)	6.47 (19.9)	8	2	15081.1501.1
									
Species group: *robusta*									
*D. robusta*	8.14	2.91–14.59		9.04 (16.1)	10.2 (14.0)	7.19 (14.0)	16	3	15020-1111.5
									
Species group: *melanica*									
*D. melanica*	5.13	3.78–5.71		5.4 (35.2)	5.71 (23.4)	5.06 (14.4)	7	4	15030-1141.3
									
Species group: *nannoptera*									
*D. acanthoptera*	16.61	8.59–19.97		19.97 (63.8)	11.95 (18.2)	9.89 (11.7)	4	4	15090-1693.0
*D. wassermani*	13.11	8.77–18.25		12 (33.1)	14.72 (24.9)	8.77 (23.2)	4	5	15090-1697.10
									
Species group: *picture wing*									
*D. grimshawi*	5.01	4.65–5.25		5.25 (60.8)	4.65 (39.2)		2	1	15287-2541.0
									
Species group: *tumiditarsus*									
*D. repletoides*	9.72	1.68–18.35		8.36 (12.8)	18.35 (12.8)	15.3 (11.9)	22	4	15250-2541.0
									
Species group: *polychaeta*									
*D. polychaeta*	3.61	2.38–4.81		3.62 (23.3)	3.33 (20.0)	3.06 (15.2)	10	4	15070-1231.0

Repeated SB of the same DNA extracts revealed very similar patterns of IGS length variation. Similarly, repeated PCR amplifications of the same DNA extracts revealed similar patterns. However, in some cases, examination of different numbers of individuals or DNA amounts of the same species resulted in slightly different patterns of IGS length variation. Nevertheless, the size index for each species was very similar across different numbers of individuals and different DNA amounts (results not shown).

### Comparison of methods

**Figure 2:**
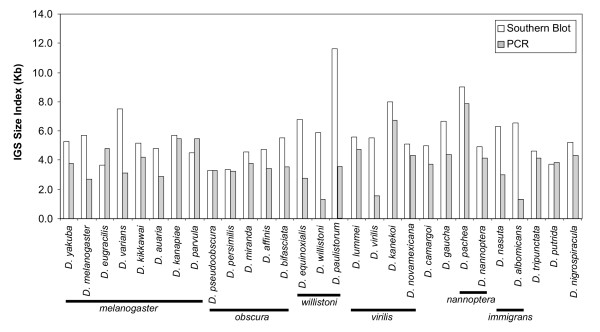
**Intergenic spacer (IGS) size index (i.e., weighted average length) based on Southern Blot hybridization and PCR in females of 29 species**. Species group to which they belong is indicated below each species group.

We compared IGS size index based on SB and PCR for the 29 species for which presence of one restriction site was confirmed (Figure 2). Average IGS length (i.e., the size index) ranged from 3.3 kb in *D. pseudoobscura *to 11.6 kb in *D. paulistorum *(Figure 2; Table 1). It is unlikely that the large fragments resulted from incomplete digestion because use of different amounts of restriction enzyme and of DNA resulted in similar patterns (results not shown). In most cases, the size index based on PCR was smaller than the one inferred from SB (Figure 2; Table 1), although in a few cases they were almost equal and in three cases (i.e., *D. eugracilis*, *D. parvula*, and *D. putrida*), the PCR-based estimates were actually larger. The difference between the size index based on SB and the one based on PCR ranged from zero in *D. pseudoobscura *to 8 kb in *D. paulistorum*.

**Figure 3:**
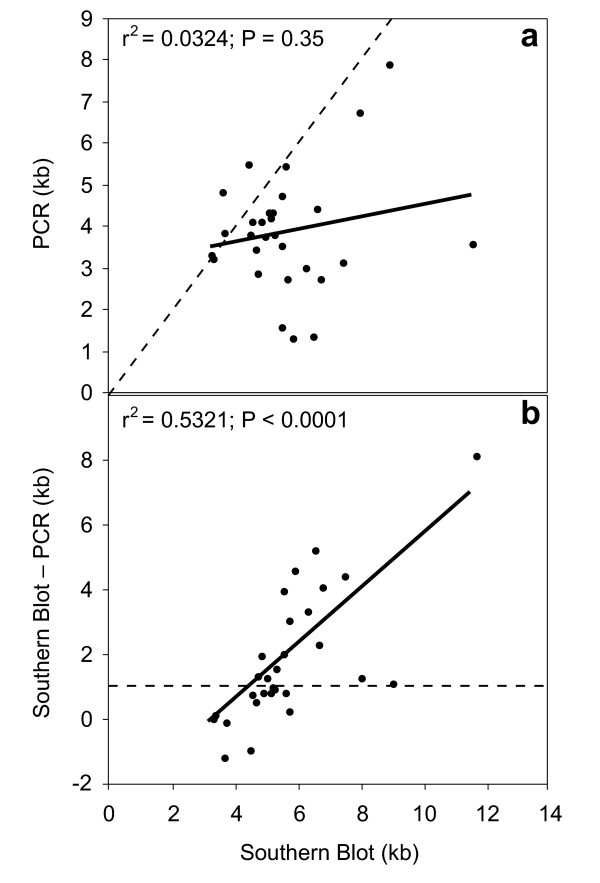
**Comparison of PCR and SB methods**. Subset of 29 species for which presence of a single restriction site within the intergenic spacer (IGS) could be confirmed (see text). **a. **Relationship between IGS size index estimated from PCR and from Southern Blot hybridization (y = 0.1465x +3.012; r^2 ^= 0.0324; *P *= 0.3498). For reference, dashed line represents equal PCR and SB values. **b. **IGS size index difference between Southern Blot hybridization and PCR vs. IGS size index based on Southern Blot hybridization. (y = 0.8535x - 3.012; r^2 ^= 0.5321; *P *< 0.0001). Dashed line indicates SB-based index minus PCR-based index = 1 kb.

To compare the results from both methods for the 29 species for which presence of the expected single restriction site was confirmed, we performed least squares regression analysis as implemented in JMP [11] of the following variables: (1) PCR-based IGS index on SB-based IGS index (Figure 3a); (2) the IGS size difference between the two methods (i.e., SB minus PCR) on SB-based IGS index (Figure 3b). Our results suggest that although in most cases PCR-based sizes were smaller than SB-based sizes (i.e., most data points fell below the dashed line; Figure 3a), no relationship exists between the IGS size index inferred from SB and that inferred from PCR (i.e., the regression is not significant). In other words, the difference between the two methods is not consistent across taxa. The differences observed between the two methods can be attributed to: (1) the possibility that the *Hae *III site No.1 (Figure 1), which occurs upstream of the forward primer (and thus, not within the PCR amplified product), was lost and therefore the IGS size based on SB was overestimated; (2) measurement error; (3) differences in the length of the sequences adjacent to the IGS that are targeted by each method (i.e., the PCR fragment is not the same as the SB fragment; see Figure 1); or (4) the tendency of the PCR to amplify smaller fragments. Given the highly conserved nature of the region where *Hae *III site No.1 is found, we do not expect this site to have been lost often within *Drosophila*. However, loss of this restriction site is a concern in the case of *D. paulistorum *due to the large difference between PCR and SB results. Although, we lack an estimate of measurement error, our results based on multiple IGS-length estimates of the same taxon with a single method never differed by more than 1 kb (see Table 1). Similarly, based on the known sequence of the regions adjacent to IGS in several *Drosophila *species, the expected difference between the PCR and SB estimates should not exceed ~200 bp. Thus, we adopt the criterion that the difference between the two methods should be greater than 1 kb (dashed line Figure 3b) for it to be regarded as a true difference due to the method used. Based on this criterion, in about half of the comparisons the PCR-based estimates were smaller than SB-based estimates; most of which had a SB-based estimate of 5 kb or more. This is further illustrated by the observation that the size difference between the two methods increased as the size based on SB increased (Figure 3b), suggesting that the larger the IGS fragment, the greater the degree of underestimation based on PCR. This relationship is still significant after removing the results from *D. paulistorum *(not shown; *P *= 0.0019). An alternative, explanation is that the PCR-based results were accurate and thus the degree of overestimation by the SB method increases as the true IGS size decreases. This is unlikely however, because in *D. melanogaster *for example, the true length of the most common variant is known based on sequence data, and PCR-inferred IGS lengths of this species were always smaller.

### IGS length variation across the genus *Drosophila*

**Figure 4:**
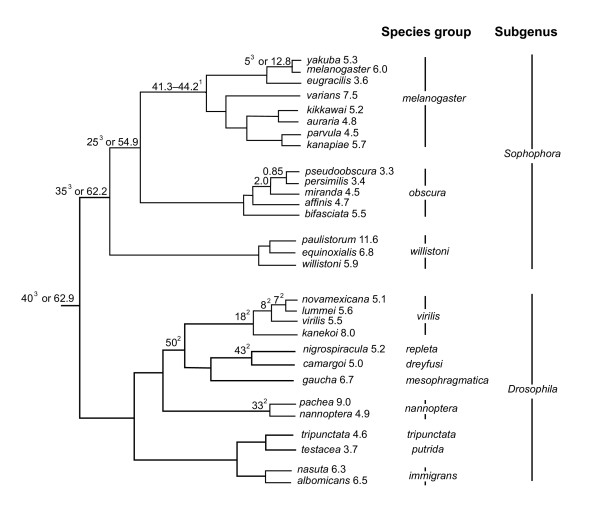
**Intergenic spacer (IGS) size index (i.e., weighted average length) based on Southern Blot hybridization in females of 29 species of the Drosophilinae subfamily**. Cladogram of phylogenetic relationships among species groups is based on Remsen and O'Grady [31]. Relationships within species groups are based on: *melanogaster *group [32]; *virilis *group [33, 34]; *obscura *group relationships [35]; and *willistoni *group [36]. Numbers above nodes indicate approximate date of divergence (in million years) based on Tamura et al. [19], unless otherwise noted. ^1 ^based on divergence of the *melanogaster *subgroup versus the *montium *and *ananassae *subgroups [19]; ^2 ^based on Pitnick et al. [18]; ^3 ^based on Russo et al. [27].

Unless otherwise noted, we discuss IGS length variation in *Drosophila *based only on the 29 species for which presence of *Hae *III site No.2 could be confirmed and for which no evidence of additional restriction sites within the IGS was observed (see above). We observed broad variation in IGS length across the genus *Drosophila*. The IGS size index ranged from 3.3 kb in *D. pseudoobscura *to 11.6 kb in *D. paulistorum *(Figures 2 and 4; Table 1). Even if we exclude *D. paulistorum *from our interpretation (see above), the IGS size index range is still broad, with *D. pachea *(9 kb) representing the species with the largest IGS index.

### Within species groups

IGS size index variation within species groups based on SB was lower than across the subfamily (Figure 4). For example, among the eight species examined from the *melanogaster *species group the largest difference between species was 3.9 kb (i.e., *D. eugracilis *vs. *D. varians*). Similarly, among the four species from the *virilis *species group, the largest difference was 3 kb (i.e., *D. novamexicana *vs *D. kanekoi*). The largest difference among five species in the *obscura *group was even smaller; 1.4 kb between *D. pseudoobscura *and *D. affinis*. The largest difference was observed in the *willistoni *species group; 5.7 kb between *D. willistoni *and *D. paulistorum*, but with the caveat that the result for *D. paulistorum *may be an overestimation (see above). The difference between *D. nannoptera *and *D. pachea *(*nannoptera *group) also was relatively large; 4.1 kb.

Comparisons between more closely related species suggest that they tend to have very similar IGS indices: *D. parvula *(4.5 kb) vs. *D. kanapiae *(5.7 kb); *D. novamexicana *(5.1 kb) vs. *D lummei *(5.6 kb); and *D. persimilis *(3.4 kb) vs. *D. pseudoobscura *(3.3 kb). The only exception was the comparison between *D. paulistorum *(11.6 kb) and *D. equinoxialis *(6.8 kb), but as mentioned above, the value for *D. paulistorum *may be an overestimation.

### Within species variation

Based on SB, all of the species except one (i.e., *D. putrida*) had more than one IGS length variant (Table 1). In most cases, the length difference between the shortest and longest IGS length variant was at least 3 kb. However, for species in which more than one individual was used, we cannot distinguish between intra- and inter-individual variation. Nevertheless, we found differences among species in the number of fragments present in species where we examined single individuals; *Drosophila robusta *and *D. melanogaster *had the highest number of bands per individual (16 and 9–15, respectively), whereas *D. grimshawi *had only two bands and *D. putrida *had only one (Table 1).

### Results based on PCR only

IGS sizes based on PCR were generally smaller than IGS sizes based on SB (Figure 2). Nevertheless, it is interesting to point out patterns of IGS size variation in the species for which SB could not be used due to the absence of one or both of the *Hae *III restrictions sites or to the presence of additional restriction sites in the IGS region. For the subset of species that were examined only by PCR, the size index ranged from 2.4 kb in *D. seguyi *and *D. birchii*, to 7.1 kb in *D. mettleri *(Table 1). Very close relatives or sister species tended to have similar lengths. For example, *D. ananassae *(4.1 kb) vs. *D. pallidosa *(4.3 kb); *D. greeni *(2.7 kb) vs. *D. seguyi *(2.4 kb); and *D. arizonae *(4.3 kb) vs. *D. mojavensis *(4.2–4.8 kb). Although these PCR-based values may not represent the true IGS size index (see below), they may provide a minimum estimate for IGS size.

### Results based on SB only

For the remaining 19 species, we only report IGS sizes based on SB because we were unable to obtain PCR product. However, these results should be considered with caution because the presence of *Hae *III site No. 2 or of additional restriction sites could not be assessed. There are several possible explanations for our inability of obtain PCR product in these species. First, it is possible that IGS fragments were not amplified because they were too large (i.e., the largest fragment we were able to amplify was 7.9 kb in *D. pachea*). It is important to note that amplification of the IGS region in *Drosophila *is not trivial because of the length (i.e., usually above 3 kb), and the high degree of secondary structure present in this region [12]. Second, the priming sites could have diverged, although our PCR primers target highly conserved regions of the 28S and 18S ribosomal genes. Finally, despite having tried a large variety of amplification conditions, we may not have found the appropriate ones for that particular species.

Based on the SB results, the largest IGS size index observed was 16.6 kb in *D. acanthoptera*. One of its relatives in the *nannoptera *group, *D. wassermani*, also had a large IGS size index, 13.1 kb. Although it is possible that these results based on SB are an overestimation of IGS size (i.e., loss of a restriction site), it is interesting to note that *D. pachea*, another member of the *nannoptera *group (for which we were able to confirm the presence of *Hae *III site No.2), also had a relatively large IGS size of 9 kb. Interestingly, the most basal member of this group, *D. nannoptera*, had a much smaller IGS index of 4.9 kb. These SB-based results also suggest broad variation in the *melanogaster *species group, with *D. bicornuta *having the largest IGS index of 10.8 kb.

## Discussion

Our study showed that in about half of the taxa examined IGS length estimates based on PCR were smaller than those estimated with SB, particularly when IGS sizes exceeded 5 kb, suggesting that PCR tends to underestimate the true IGS length because of selective amplification of smaller fragments. A comparison with results from previous studies suggests this. For example, studies of IGS length variation in *D. melanogaster *based on SB show that this species has many fragments larger than 5 kb [5,13]. In contrast, the studies that used PCR to infer IGS length in this species found that amplified fragments were always smaller than 4 kb [9,10]. Therefore, SB seems to be the most appropriate method for IGS length inference. However, knowledge about the sequence is required, or at least the presence of the appropriate restriction sites on the ends of the fragment of interest should be confirmed. For example, in the present study, we were able to confirm the presence of one of these restriction sites (i.e., *Hae *III site No. 2), by PCR amplification of IGS, followed by restriction digestion of PCR products. Nevertheless, the PCR fragment ideally should span the region that contains both restriction sites, because in at least one case (i.e., *D. paulistorum*), we suspect the other restriction site may have been lost. Unfortunately, we were unable to obtain amplification with PCR primers that spanned the region that contained both restriction sites.

### IGS size variation

Our study revealed that IGS size index variation among species of the subfamily Drosophilinae is broader than previously reported [5,13-17]: from 3.3 kb in *D. pseudoobscura *to 9 kb in *D. pachea *and possibly 11.6 kb in *D. paulistorum*. Considering that *D. pseudoobscura *diverged from *D. pachea*, 40–63 million years ago, and from *D. paulistorum *35–62 million years ago (Figure 4), the large IGS length differences are not surprising; particularly in light of the observation that average IGS length has been demonstrated to change rapidly after artificial selection in *D. melanogaster *[i.e., 24 generations to shift the average size from 5.54 to 5.8 kb and 15 generations to shift the average size from 5.54 to 5.12 kb; [5]].

Despite the speed at which IGS has been shown to evolve under selection, comparisons between very closely related taxa, including sister species pairs, suggest that they tend to have very similar IGS indices. For example, the close relatives, *D. parvula *and *D. kanapiae *differ from each other by 1.2 kb; and *D. novamexicana *and *D. lummei *[i.e., ~6 million-year-divergence; [18]] differ by 500 bp. An even more closely related species pair [i.e., ~ 0.85 million-year-divergence; [19]], *D. persimilis *and *D. pseudoobscura*, differ only by 100 bp. The only exception was the comparison between *D. paulistorum *and *D. equinoxialis*, who differ by 4.8 kb, but as mentioned above, the value for *D. paulistorum *may be an overestimation. Although the PCR-based results should be interpreted with caution, they may offer additional insight regarding patterns of IGS length across closely related taxa. For example, *D. ananassae *differs from its close relative *D. pallidosa *by 200 bp; *D. greeni *differs from *D. seguyi *by 300 bp; and *D. lini *differs from *D. kikkawai *(based on PCR) by 800 bp. Finally, the IGS index of *D. arizonae *is within the range of values reported for two populations of its sister species *D. mojavensis*, from which it diverged approximately 1–1.2 million years ago [20,21].

Comparisons of more distantly related taxa, even within the same species group show less clear patterns. For example, the four members of the *montium *subgroup of the *melanogaster *species group examined in this study (i.e., *D. kikkawai*, *D. auraria*, *D. parvula*, and *D. kanapiae*) differ from each other by a maximum of 1.2 kb. On the other hand, based on our results and the ones of Coen et al. [14], IGS size index ranges from 3.6 to 6 kb among eight members of the *melanogaster *subgroup (*D. melanogaster*, *D. mauritiana*, *D. simulans*, *D. erecta*, *D. yakuba*, *D. teissieri*, *D. orena*, and *D. eugracilis*) another subgroup within the *melanogaster *species group. Similarly, based on our results and the ones from Rae et al. [17], IGS size index ranges from 4.2 to 8.0 kb across nine members of the *virilis *species group; thus, the largest IGS index observed in this group almost doubles the smallest one. A large difference (i.e., 4.1 kb) is also observed between *D. pachea *and *D. nannoptera *(*nannoptera *group). Nevertheless, despite being members of the same species group, these two taxa may be up to 32 million years divergent [18], providing a long period for the accumulation of such differences. Unfortunately, similar comparisons of IGS length and divergence time are not possible for many of the taxa examined in this study because we lack divergence time estimates.

The observation that close relatives tend to have similar IGS indices, whereas more distant relatives may not, is consistent with the observation that closely related taxa exhibit a high degree of DNA sequence homology of the IGS region [as observed among members of the *melanogaster *subgroup; [22]], whereas more distantly related taxa exhibit no DNA sequence homology [as reported between the subgenera *Sophophora *and *Drosophila*; [16,23]], despite showing similarities in structure such as promoter duplications.

The ecological and evolutionary implications of the broad variation in IGS size observed across members of the Drosophilinae are largely unknown. However, several evolutionary mechanisms appear to play a role in the evolution of IGS variation. First, as a member of the ribosomal DNA multigene family, IGS is subject to concerted evolution [24]. The pattern of concerted evolution appears to be the result of unequal crossing over taking place both, at the level of the subrepeat arrays within the intergenic spacers, and at the level of the complete rDNA units (i.e., genes plus spacers) [25]. The former would create new IGS length variants while the latter would spread a particular variant across the chromosome(s). In addition, the high within-species specificity of the RNA polymerase I complex [26] suggests that IGS coevolves with components of transcriptional machinery. Finally, individual IGS variants may be adaptive, particularly with regard to developmental rate, as suggested by studies of two unrelated taxa, *Drosophila melanogaster *[5] and *Daphnia pulex *[6,8]. This observation has led to the suggestion that IGS length alone makes a considerable contribution to growth rate differences and hence life history evolution among related species [6]. Although in long evolutionary time scales, IGS length is highly variable across *Drosophila*, it does not appear vary broadly in shorter time scales. However, examination of other species may reveal additional variation in shorter time scales that may provide the necessary variation for testing this hypothesis. Nevertheless, tests of this hypothesis will only be informative if IGS length is accurately measured. Furthermore, even if IGS length is found to be adaptive, the crucial assumption that IGS length represents the number of promoter copies should ultimately be tested by DNA sequencing.

## Conclusion

Broad variation in average IGS length occurs across large evolutionary scales in members of the subfamily Drosophilinae. However, despite the potential for rapid changes in IGS length shown by artificial selection studies, closely related taxa tend to have similar IGS sizes. Our comparison of methods suggests that PCR-based estimations tend to underestimate the true IGS size when the IGS size is greater than 5 kb and thus, in the absence of DNA sequence information for all the taxa under examination, both methods should be used.

## Methods

### Taxon selection

To examine the extent of IGS length variation across the subfamily Drosophilinae, where possible, we examined at least one species per major species group. Our taxon sampling scheme spanned divergences of at least 40 [27] or 63 [19] million years based on the estimated average divergence between members of the subgenus *Sophophora *and the subgenus *Drosophila *(genus *Drosophila*). To assess the amount of IGS length variation present in shorter evolutionary time scales, we examined closely related species, including sister species pairs.

### Southern Blot

We extracted DNA from 1–10 individual female flies per species. We only examined female flies to prevent any sex bias in our interpretation. In *D. melanogaster*, and a few other species, Nucleolar Organizer Regions (NORs) are found on the X and Y chromosomes but in other species the locations are not entirely clear [28,29]. Furthermore, previous studies have shown that the Y-linked IGS variants of *D. melanogaster *are not related to differences in development time [13]. Whole flies were homogenized in 250 μl of DNAzol (Invitrogen, Carlsbad, CA) and 0.1 mg of Proteinase K, and incubated overnight at room temperature. Following centrifugation to discard cellular debris, DNA was precipitated by addition of 125 μl of 100% ethanol and overnight incubation at -20°C. The DNA pellet was recovered by centrifugation, then washed twice with 70% ethanol. DNA was resuspended in 20–100 μl of sterile deionized water and incubated 2–3 hr at 65°C.

We digested DNA extracts overnight with *Hae *III (New England Biolabs (Beverly, MA) following manufacturer's instructions, and treated with 0.4 μg/μl RNAse A for 5 min at room temperature. The *Hae *III enzyme was selected because *Hae *III sites are found on either end of the IGS in *Drosophila melanogaster *(Figure 1), and its distant [i.e., 40–63 million years divergent; [19,27]] relative *D. virilis *[17]. *Hae *III site No. 1 is also present in a distant relative of *D. melanogaster*, *D. hydei*, but sequences of the 3' end of IGS are lacking for this and other species, so presence of *Hae *III site No. 2 has not been confirmed. We then treated the digested DNA with SDS to a final concentration of 0.1% and proteinase K to a final concentration of 20 μg/ml followed by a 30 min incubation at 37°C. This treatment improves the migration of DNA during electrophoresis by removal of contaminating protein [30].

Samples were electrophoresed on 0.9% agarose gels and blotted onto positively charged Nylon membranes (Roche Applied Science, Indianapolis, IN) with the VacuGene XL (Amersham Biosciences Corp, Piscataway, NJ) according to manufacturer's instructions. DNA on the membrane was then UV crosslinked with the Stratalinker (Stratagene, Cedar Creek, TX) according to manufacturer's instructions.

We used a ~300 bp portion of the highly conserved 3' end of the 28S gene as a hybridization probe (Figure 1). We first amplified the fragment of interest in a solution containing ~1 μl of template in a final concentration of 1% DMSO, 20% Betaine, 0.2 mM dNTPs, 5 mM MgCl, 0.2 μM primers (28S-R3665 5'-TTATTTATCATTGCAGTCCAGCACGG-3' and 28S-F3349 5'CATAGCGACGTCGCTTTTTGATCC-3'), 2 units of *Taq *Polymerase (Invitrogen, Carlsbad, CA) and 1X of Buffer provided by manufacturer. The PCR template contained a mix of genomic DNA from one species per species group examined. The temperature profile had an initial denaturation of 2 min at 95°C, followed by 35 cycles of 1 min at 95°C, 1 min at 58°C and 1 min at 72°C, and a final extension of 7 min at 72°C. The amplified product was electrophoresed on an agarose gel and the fragment of interest was excised and used as template for an asymmetric PCR. This reaction was identical to the first one with the exception that we used less 28S-R3665 primer (final concentration of 0.002 μM) and we substituted regular dNTPs with those contained in the DIG DNA Labeling Mix (Roche Applied Science, Indianapolis, IN) to a final concentration of 0.4 mM. The labeled product was purified by Ethanol precipitation with Sodium Acetate, resuspended in ~100 μl, and added to hybridization buffer (below).

Pre-hybridization, hybridization, and high stringency washes were performed in a hybridization oven. All other incubation/washes were performed with slight agitation. Hybridization and washing solutions were prepared from two stock buffers: 20X SSC (3M NaCl, 300 mM sodium citrate, adjust with Citric Acid to pH 7.0) and 1X Maleic Acid (0.1M Maleic Acid, 0.15M NaCl adjusted with NaOH to pH 7.5). We incubated blotted membranes 2 hr at 68°C in prehybridization buffer [5X SSC; 2% (w/v) blocking reagent (Roche Applied Science, Indianapolis, IN) dissolved by heating; 0.1% N-lauroylsarcosine; and 0.02% SDS (w/v)]. We then incubated membranes overnight at 68°C in hybridization buffer (same as prehybridization buffer plus probe). Hybridization buffer (with probe) was boiled for at least 10 min prior to incubation. We washed hybridized membranes (five 5-min washes at room temperature) with a low stringency buffer (2X SSC containing 0.1% SDS). We then washed membranes (three 10-min washes at 68°C) with a high stringency buffer (0.1X SSC containing 0.1% SDS). Membranes were then equilibrated 2 min in washing buffer (1X Maleic Acid; 0.3% (v/v) Tween 20). We incubated membranes 45 min at room temperature in blocking solution (2% (w/v) blocking reagent in 1X Maleic Acid; dissolved by heating). We then incubated membranes 45 min at room temperature in Antibody solution (i.e., blocking solution and 1:10,000 Anti-Digoxigenin-AP, Roche Applied Science, Indianapolis, IN). Membranes were washed (two 10-min washes) in washing buffer and equilibrated (2–5 min) in detection buffer (0.1M Tris-HCl, 0.1m NaCl, pH 9.5). We added the chemiluminescent substrate CSPD (Roche Applied Science, Indianapolis, IN) following manufacturer's protocol and exposed the membrane to Kodak Biomax light-1 X-ray film for 15–180 min).

### Analysis

X-rays were photographed with a Kodak Edas 290 digital camera and analyzed with Kodak 1D 360 software to determine molecular weight of each band observed as well as its relative intensity (with respect to other bands in the same lane). We use relative intensity as a proxy of relative copy number of each band.

We estimated the weighted average spacer length index (*I*) for each lane based on the length (i.e., molecular weight) and proportion (i.e., relative intensity) of each band as in Cluster et al. [5]:



where *n *is the number of spacer bands in a lane, *S*_*i *_is the fragment size (or molecular weight) of each band, and *P*_*i *_is the relative intensity. *S*_*i *_was estimated by comparison with standards.

### PCR amplification of IGS

To evaluate the consistency of PCR and Southern Blot hybridization (SB) in estimation of IGS length, we amplified the IGS region from females of the same strains examined by SB. Our PCR reactions (25 μl total volume) contained ~2 μl of template in a final concentration of 1% DMSO, 20% Betaine, 0.4 mM each dNTP, 3 mM MgCl, 0.2 μM primers IGSF2 5'-GTGCTGGACTGCAATGATAAATAAGG-3' (K. Glenn, unpublished) and IGSR1 5'-AAGCATATAACTACTGGCAGGATCAACC-3' (Y-C. Li, unpublished), 2 units of *Taq *Polymerase (Invitrogen, Carlsbad, CA) and 1X of Buffer provided by manufacturer. The IGSF2 primer is located in a conserved region at the 3'-end of the 28S gene; approximately 300 bp downstream of *Hae *III site No. 1 in two distantly related species [i.e., *D. melanogaster*; 23 and *D. hydei*; GenBank Acc. Nos. M21017 and AF465783, respectively; see Figure 1]. The IGSR1 primer is located in a relatively conserved region of the 18S gene; approximately 200 bp downstream of *Hae *III site No. 2 in *D. melanogaster *and *D. virilis *[23]; two distantly related species. Therefore, the amplified IGS fragments were expected to have a single restriction site near the 3'end. Following PCR, half of the amplified product was treated with *Hae *III to establish whether *Hae *III sites existed within the PCR amplified fragments. The *Hae *III-treated and untreated PCR products were run side by side on 1% agarose gels.

## Authors' contributions

MM designed and conducted the experiments and analyses, and drafted the manuscript. TAM conceived the study, and participated in its design and coordination and helped to draft the manuscript. Both authors read and approved the final manuscript.
